# Racial, sex, and geographic disparities in mortality involving stroke and chronic kidney disease: a CDC WONDER database analysis, 1999–2020

**DOI:** 10.1097/MS9.0000000000005695

**Published:** 2026-09-10

**Authors:** Abhishek Kumar Shah, Suhana Chaulagain

**Affiliations:** a Maharajgunj Medical Campus, Institute of Medicine, Tribhuvan University Teaching Hospital, Kathmandu, Nepal; b Patan Academy of Health Sciences, Lalitpur, Nepal

**Keywords:** chronic kidney disease, disparities, mortality, stroke

## Abstract

**Introduction:**

Chronic kidney disease (CKD) increases the risk of stroke, and disparities exist in related mortality. Few studies have examined the mortality patterns involving both conditions together. This study evaluates national trends and disparities in stroke- and CKD-related mortality in the United States from 1999 to 2020.

**Methods:**

We analyzed death certificate data from the Centers for Disease Control and Prevention Wide-Ranging Online Data for Epidemiologic Research (CDC WONDER) database using ICD-10 codes I60–I69 for stroke and N18 for CKD. Age-adjusted mortality rates (AAMRs) per 100 000 population were calculated and stratified by sex, race/ethnicity, age group, census region, and urban–rural classification. Temporal trends were analyzed using Joinpoint regression to estimate annual percent changes with 95% confidence intervals.

**Results:**

Between 1999 and 2020, a total of 140 501 stroke- and CKD-related deaths were recorded. The overall AAMR declined from 3.42 in 1999 to 2.71 in 2020. Females had the most deaths, but males had slightly higher AAMRs. The Black population exhibited the highest AAMR. Mortality increased with age and was highest among those aged 85 years or older. Geographic disparities were evident, with the highest rates in the southern states (the Stroke Belt).

**Conclusion:**

Mortality from stroke and CKD has declined over two decades, but disparities still persist across race and ethnicity, geography, and sex. Targeted prevention and healthcare access are essential to reduce these disparities.

## Introduction

Chronic kidney disease (CKD) is a strong risk factor for stroke, which remains a major cause of illness and death globally. Individuals on dialysis have a tenfold greater risk of stroke, with mortality rates as high as 90%^[^1^]^. In addition to increasing the incidence of stroke, CKD also worsens outcomes after stroke. This elevated risk is driven by both traditional cardiovascular risk factors, such as hypertension, diabetes, and dyslipidemia, and CKD-specific nontraditional risk factors, including albuminuria, fluid overload, inflammation, and vascular calcification^[^2^]^.

HIGHLIGHTS

- Stroke- and CKD-related mortality declined in the United States from 1999 to 2020.
- Overall, males had higher age-adjusted mortality rates than females.
- Black Americans had the highest mortality but experienced the greatest decline.
- Southern “Stroke Belt” states recorded the nation’s highest AAMR levels.
- Disparities by race, sex, and geography persist despite overall progress.

Substantial disparities exist in stroke and CKD outcomes across demographic groups. Higher incidence and mortality rates are observed in Black and Hispanic populations compared to White individuals^[^3^]^. Similarly, men often exhibit higher mortality from stroke, while women tend to have a higher prevalence of CKD^[^4,5^]^. The “Stroke Belt” states in the southeastern US have disproportionately high mortality rates, thus showing geographic differences^[^6^]^. These disparities suggest the influence of social determinants of health, unequal access to care, and variations in healthcare quality.

While numerous studies have examined racial and sex disparities in stroke or CKD when considered separately, few have explored mortality patterns involving both conditions simultaneously^[^7,8^]^. Moreover, most previous analyses of cardiovascular mortality among patients with CKD have been restricted to earlier years or to specific regions or age groups, thereby limiting the understanding of nationwide trends. Importantly, there are no studies that address stroke-specific outcomes in this population. With the availability of the Centers for Disease Control and Prevention Wide-Ranging Online Data for Epidemiologic Research (CDC WONDER) database, long-term, population-level trends in mortality can now be systematically assessed by demographic and geographic factors. This study aimed to evaluate racial, sex, and geographic disparities in mortality involving stroke and CKD in the United States from 1999 to 2020 using the CDC WONDER database.

## Methods

### Study setting and data source

A retrospective analysis of stroke- and CKD-related mortality was conducted using data obtained from the CDC WONDER database. The study period comprised data collected between 1999 and 2020. Mortality from 1999 to 2020 was examined using codes I60–I69 for stroke and N18 for CKD from the International Statistical Classification of Diseases and Related Health Problems, Tenth Revision (ICD-10). Multiple cause-of-death public-use records were examined to identify deaths in which stroke or CKD was listed as either the underlying or a contributing cause of death, and these records have been used in prior studies^[^9^]^. Individuals with both conditions listed as either the primary cause of death or a contributing factor were included in our analysis. This study was exempt from local institutional review board approval because it used a publicly available, de-identified, government-issued dataset. The analysis and reporting followed the Strengthening the Reporting of Observational Studies in Epidemiology (STROBE) and TITAN guidelines^[^10,11^]^.

### Data extraction

The records were extracted from the database using ICD-10 codes I60–I69 for stroke and N18 for CKD and were stratified by year, population size, absolute number of deaths, gender, race/ethnicity, 10-year age groups, states, urban-rural status, census region, and place of death. The genders included were male and female, as recorded on the death certificates. Race/ethnicity was categorized as American Indian or Alaska Native, Asian or Pacific Islander, Hispanic or Latino, Black or African American, and White. The 10-year age groups were predefined as 35–44, 45–54, 55–64, 65–74, 75–84, and 85+ years.

This information depends on data reported on death certificates and has been used in prior analyses of the CDC-WONDER database^[^12^]^.

Urban–rural classification followed the 2013 US Census and the National Center for Health Statistics (NCHS) definitions, categorized as large central metropolitan, large fringe metropolitan, medium metropolitan, small metropolitan, micropolitan, and noncore. These urbanization groups were consolidated into two urban–rural tiers: metropolitan (small metro, medium metro, large fringe metro, and large central metro) and nonmetropolitan (micropolitan and noncore). According to the US Census Bureau definitions, the census regions were defined as Northeast, South, West, and Midwest^[^13^]^.

### Statistical analysis

Nationwide trends in stroke- and CKD-related mortality were examined using crude mortality rates (CMRs) and age-adjusted mortality rates (AAMRs), calculated per 100 000 population from 1999 to 2020. Rates were stratified by year, gender, race/ethnicity, 10-year age groups, place of death, state, urban-rural status, and census regions, with corresponding 95% confidence intervals (CIs). CMRs were calculated by dividing the number of deaths attributed to stroke or CKD by the respective US population for that year. AAMRs were standardized to the year 2000 US standard population^[^14^]^. The Joinpoint Regression Program (Joinpoint version 5.4.0, National Cancer Institute; produced by the Statistical Research and Applications Branch, National Cancer Institute) was used to assess trends in AAMRs and CMRs^[^15^]^. This method identifies statistically significant changes in mortality trends over time by fitting log-linear regression models and detecting points at which trends shift in direction or magnitude. Annual percent changes (APCs) with 95% CIs were calculated for each time segment at joinpoints using the Monte Carlo permutation test. APC trends were classified as increasing or decreasing when the slope of the regression line was significantly different from zero, as determined by two-tailed *t*-tests. A *P*-value <0.05 was considered statistically significant.

## Results

There were a total of 140 501 deaths due to stroke and CKD (Fig. 1 and Supplemental Digital Content Table S1, available at: https://links.lww.com/MS9/B380). Of the 140 501 deaths, data on place of death were available for 140 144 deaths. Of these, 47.51% of deaths occurred in a medical facility (inpatient), 25.83% occurred in nursing home/long-term care, 15.01% in the decedent’s home, 4.65% in a hospice facility, 3.23% in a medical facility (outpatient or emergency room), 3.14% in other locations, 0.21% in a medical facility (dead on arrival), 0.19% in an unknown location, and 0.12% in a medical facility (status unknown) (Supplemental Digital Content Table S2, available at: https://links.lww.com/MS9/B380).

### Annual trends for stroke and CKD-related AAMR

The overall AAMR for stroke- and CKD-related deaths was 3.42 in 1999 and 2.71 in 2020 (Supplemental Digital Content Table S3, available at: https://links.lww.com/MS9/B380). The trend in AAMR shows a reduction in mortality from 1999 to 2020, with an APC of −1.09 (95% CI: −2.75 to 0.58) (Fig. 2).

**Figure 1.**
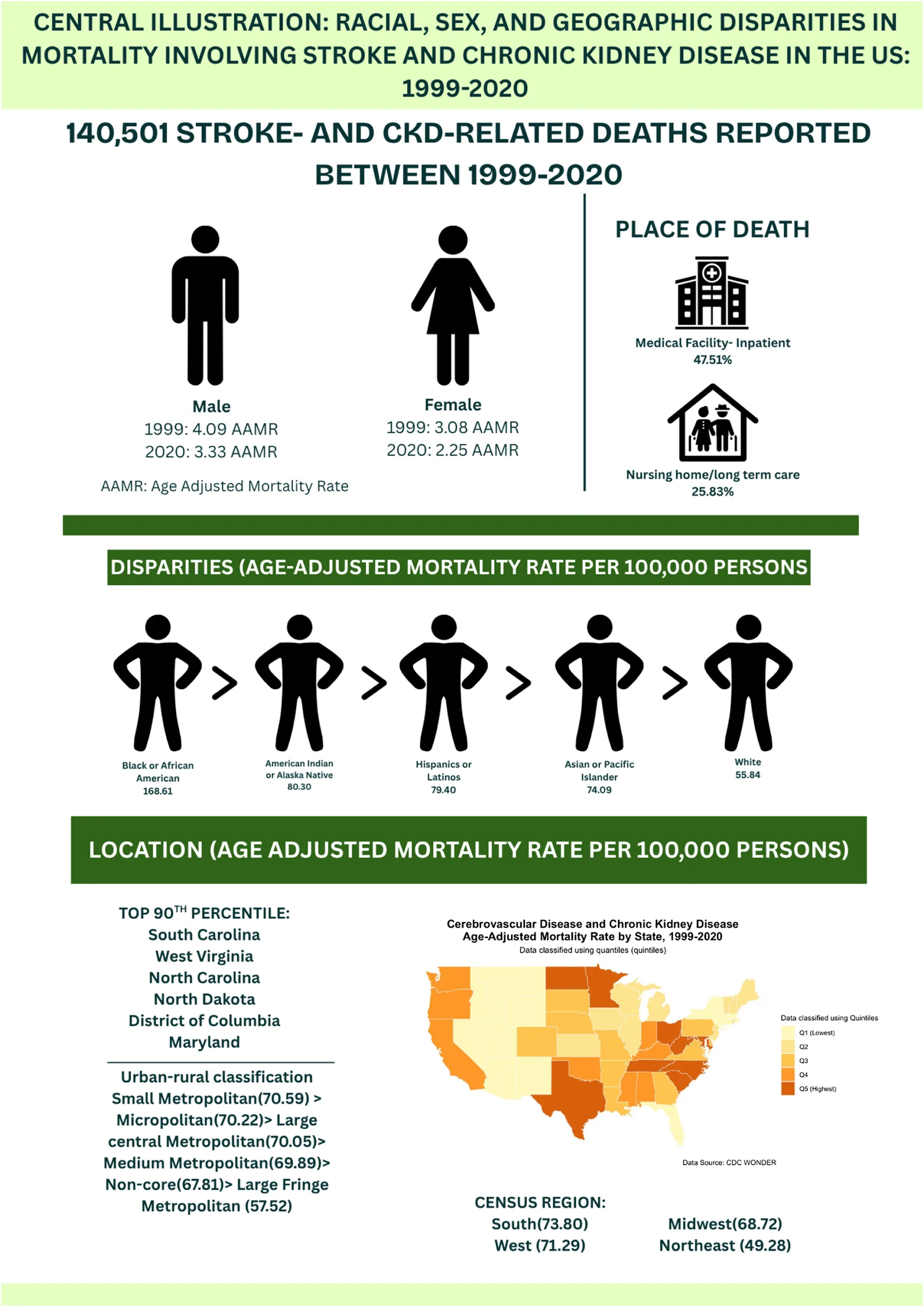
Central illustration.

**Figure 2.**
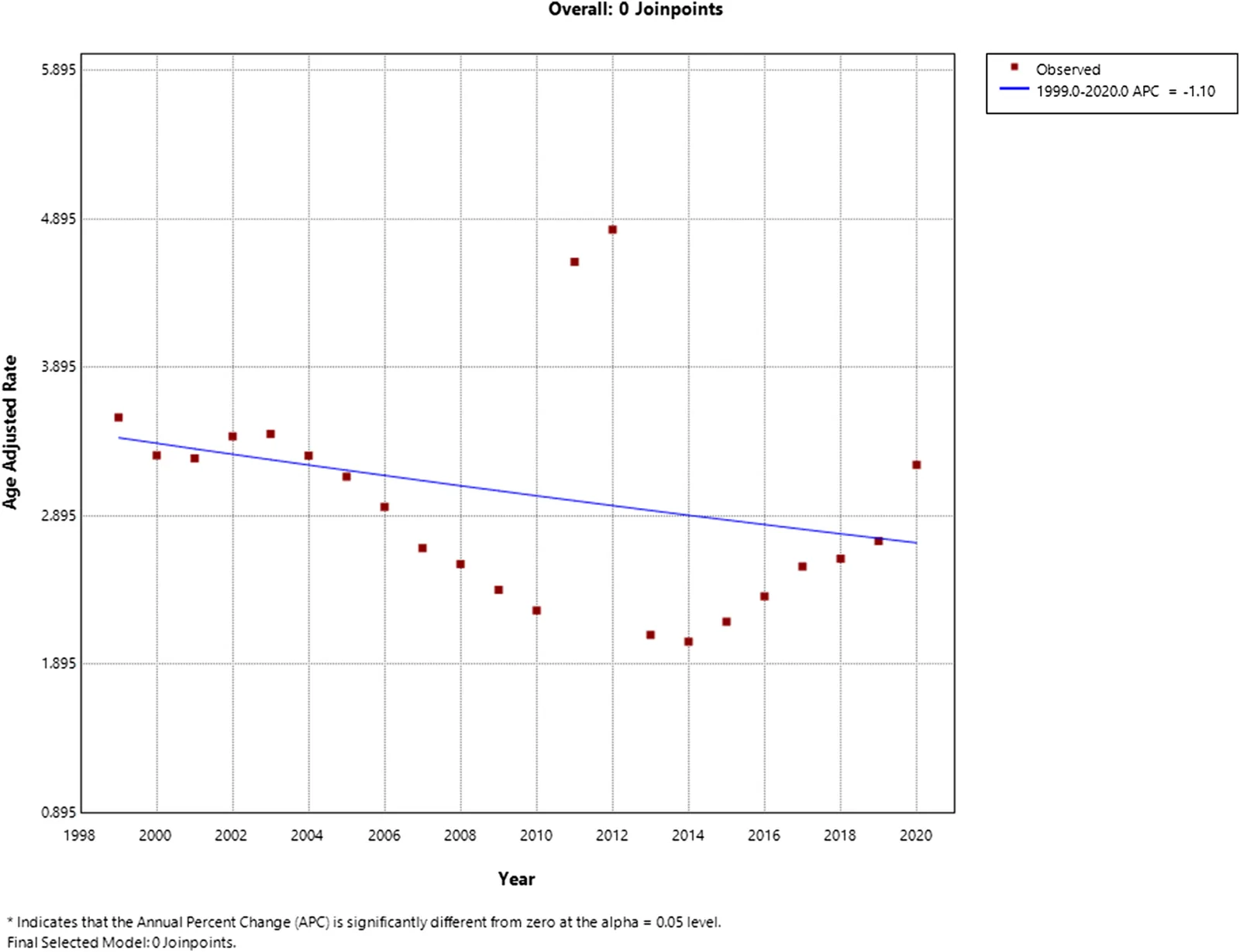
Trends in stroke- and chronic kidney disease-related overall age-adjusted mortality rates in the United States, 1999–2020.

### Stroke and CKD-related AAMR stratified by gender

The majority of deaths occurred in females, with a total of 71 177, compared with 69 324 in males (Supplemental Digital Content Table S1, available at: https://links.lww.com/MS9/B380). From 1999 to 2020, AAMR in males showed an overall decrease, with an APC of −0.98 (95% CI: −2.61 to 0.67) (Fig. 3). The AAMR was 4.09 in 1999 and 3.33 in 2020 (Supplemental Digital Content Table S3, available at: https://links.lww.com/MS9/B380). In females as well, there was a downward trend from 1999 to 2020, with an APC of −1.48 (95% CI: −3.16 to 0.21) (Fig. 3). The AAMR was 3.08 in 1999, and in 2020 it was 2.25 (Supplemental Digital Content Table S3, available at: https://links.lww.com/MS9/B380).

**Figure 3.**
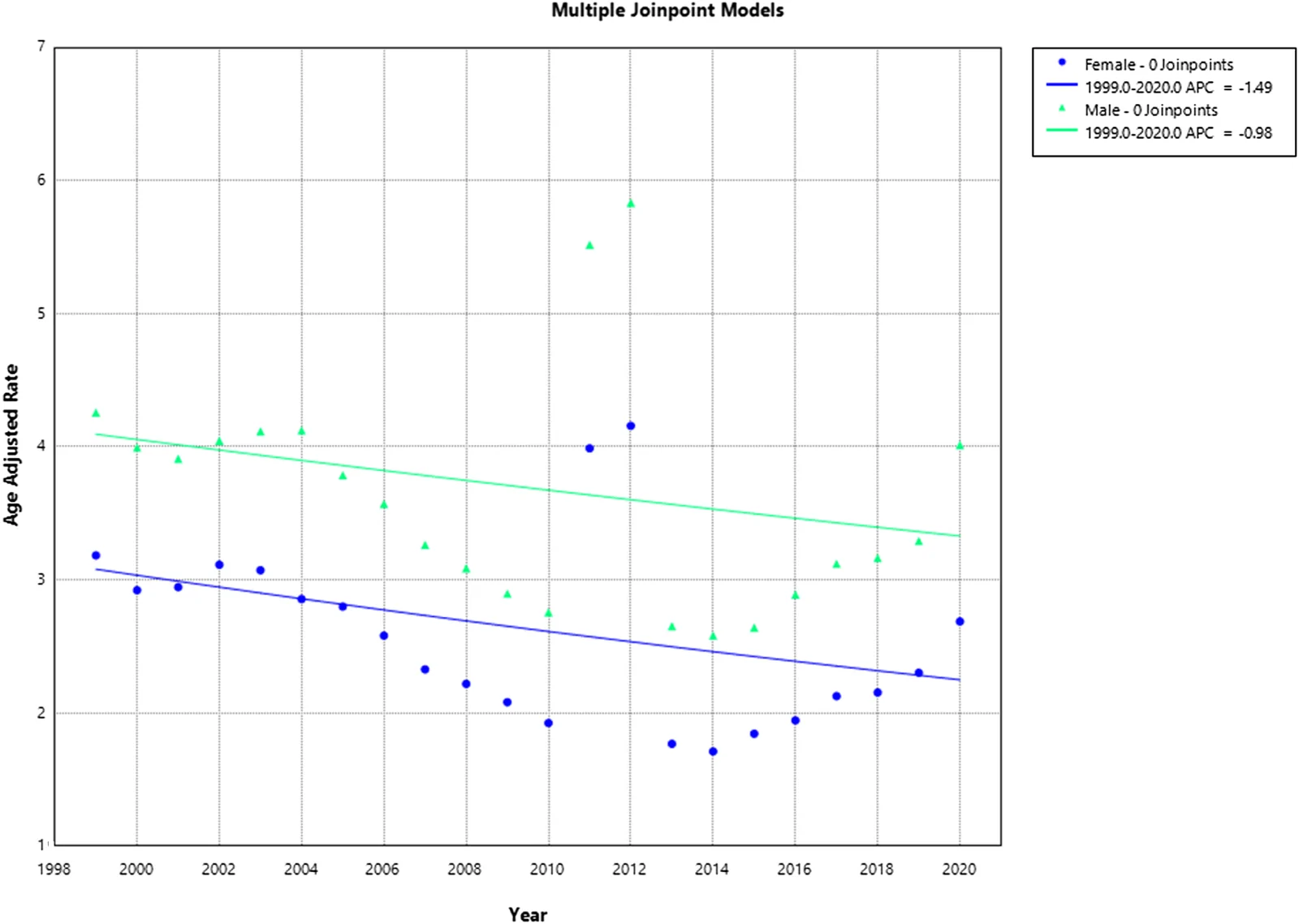
Sex-stratified trends in age-adjusted mortality rates (AAMRs) per 100 000 individuals for stroke and chronic kidney disease in the United States from 1999 to 2020.

### Stroke and CKD-related AAMR stratified by race/ethnicity

Our results show that the absolute number of deaths from 1999 to 2020 was highest in the White population (102 231), followed by the Black or African American population (31 897), Hispanic or Latino (11 507), Asian or Pacific Islander (5303), and American Indian or Alaska Native (1070) (Supplemental Digital Content Table S1, available at: https://links.lww.com/MS9/B380). The AAMR for American Indian or Alaska Native individuals generally declined from 1999 to 2020 (APC: −2.80; 95% CI: −4.68 to −0.87) (Fig. 4). They had an AAMR of 4.84 in 1999 and 2.67 in 2020 (Supplemental Digital Content Table S4, available at: https://links.lww.com/MS9/B380).

**Figure 4.**
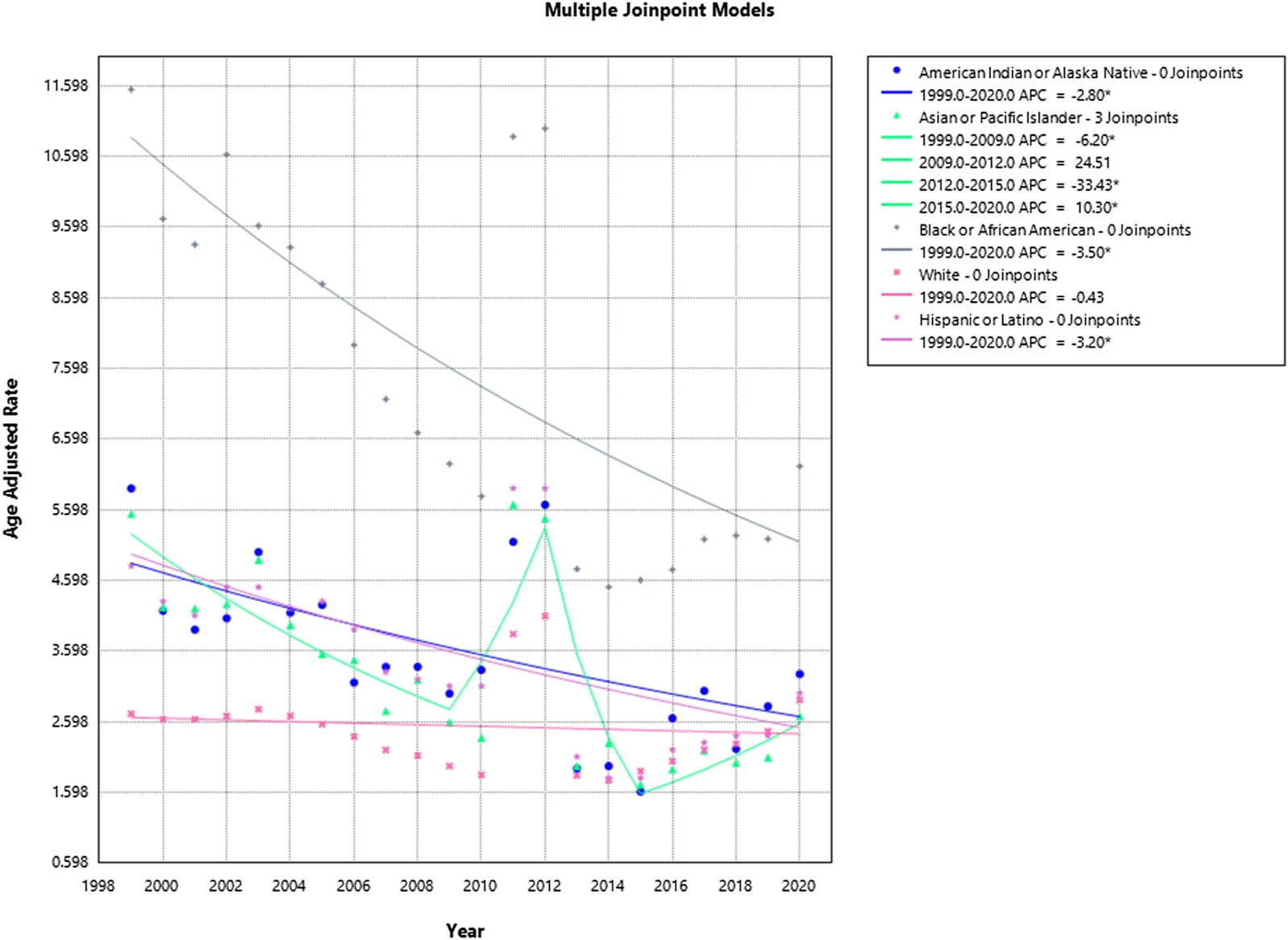
Race- and ethnicity-stratified trends in age-adjusted mortality rates (AAMRs) per 100 000 individuals for stroke and chronic kidney disease in the United States from 1999 to 2020.

The Asian or Pacific Islander population had an AAMR of 5.25 in 1999 and 2.57 in 2020 (Supplemental Digital Content Table S4, available at: https://links.lww.com/MS9/B380). Trend analysis for the Asian or Pacific Islander population’s AAMR showed a significant decline between 1999 and 2009, with an APC of −6.20 (95% CI: −8.71 to 3.62), followed by an abrupt and sharp increase in the next short segment from 2009 to 2012 (APC: 24.50; 95% CI: −8.12 to 68.73). Following this peak, the rate experienced its steepest decline between 2012 and 2015 (APC: −33.43; 95% CI: −52.79 to −6.13), and then the rate began to rise again with an APC of 10.30 (95% CI: 3.38–17.68) (Fig. 4).

In the Hispanic or Latino population, the AAMR was 4.97 in 1999 and 2.51 in 2020 (Supplemental Digital Content Table S4, available at: https://links.lww.com/MS9/B380). The AAMR decreased from 1999 to 2020, with an APC of −3.19 (95% CI: −5.19 to −1.16). Moreover, in the White population, the AAMR was 2.66 in 1999 and 2.42 in 2020. There was a very slight decrease in the age-adjusted rate from 1999 to 2020, with an APC of −0.43 (95% CI: −2.10 to 1.26) (Fig. 4).

Finally, the graph for the Black or African American population over the period from 1999 to 2020 shows that the age-adjusted rate has declined substantially over the two decades, with an APC of −3.50 (95% CI: −5.04 to −1.93) (Fig. 4). The AAMR was 10.86 in 1999 and 5.14 in 2020 (Supplemental Digital Content Table S4, available at: https://links.lww.com/MS9/B380).

### Stroke and CKD-related CMR stratified by 10-year age groups

The graph for individuals aged 35–44 years displays a statistically significant decreasing trend in the Crude Rate over the entire period from 1999 to 2020, with APC: −2.58 (95% CI: −4.29 to −0.84) (Fig. 5). They had the lowest CMR of 0.30 in 1999 and 0.17 in 2020 (Supplemental Digital Content Table S5, available at: https://links.lww.com/MS9/B380). There is a statistically significant overall decreasing trend in the Crude Rate for the 45–54-year age group over the period from 1999 to 2020 (APC: −2.17; 95% CI: −4.06 to −0.25) (Supplemental Digital Content Table S5, available at: https://links.lww.com/MS9/B380). They had a CMR of 0.89 in 1999 and 0.56 in 2020 (Fig. 5). The CMR for the 55–64 age group initially declined for about a decade (1999–2009) with an APC of −5.71 (95% CI: −6.83 to −4.56), followed by a surge from 2009 to 2012 (APC: 17.36; 95% CI: 1.70–35.43), an equally sharp collapse from 2012 to 2015 (APC: −27.77; 95% CI: −39.77 to −13.38), and a subsequent moderate upward trend from 2015 to 2020, with an APC of 11.56 (95% CI: 7.69–15.56) (Fig. 5). They had a CMR of 3.23 and 1.89 in 1999 and 2020, respectively (Supplemental Digital Content Table S5, available at: https://links.lww.com/MS9/B380). The 65–74-year age group had a CMR of 8.90 in 1999 and 5.09 in 2020 (Supplemental Digital Content Table S5, available at: https://links.lww.com/MS9/B380). They initially experienced a significant decade-long decline (1999–2009) with an APC of −5.31 (95% CI: −6.33 to −4.28), which was abruptly followed by an increase from 2009 to 2012 (APC: 13.97; 95% CI: −0.88 to 31.05), and then an equally swift and highly significant collapse from 2012 to 2015 (APC: −26.94; 95% CI: −38.15 to −13.70). The period concluded with a moderate, yet statistically significant, recovery and upward trend between 2015 and 2020, registering an APC of 11.30 (95% CI: 7.89–14.82) (Fig. 5). The data for the 75–84-year age cohort reveal a single, consistent trend across the entire period studied from 1999 to 2020, with an APC of −1.32 (95% CI: −2.93 to 0.32) (Fig. 5). This population had the second-highest CMR of 17.11 in 1999 and 12.94 in 2020 (Supplemental Digital Content Table S5, available at: https://links.lww.com/MS9/B380). The CMR for the 85+ years age group was highest in 1999 at 31.20 and in 2020 at 44.55 (Supplemental Digital Content Table S5, available at: https://links.lww.com/MS9/B380). A moderate upward trend over the entire period, from 1999 to 2020, with an APC of 1.71 (95% CI: −0.21 to 3.67) was observed (Fig. 5).

**Figure 5.**
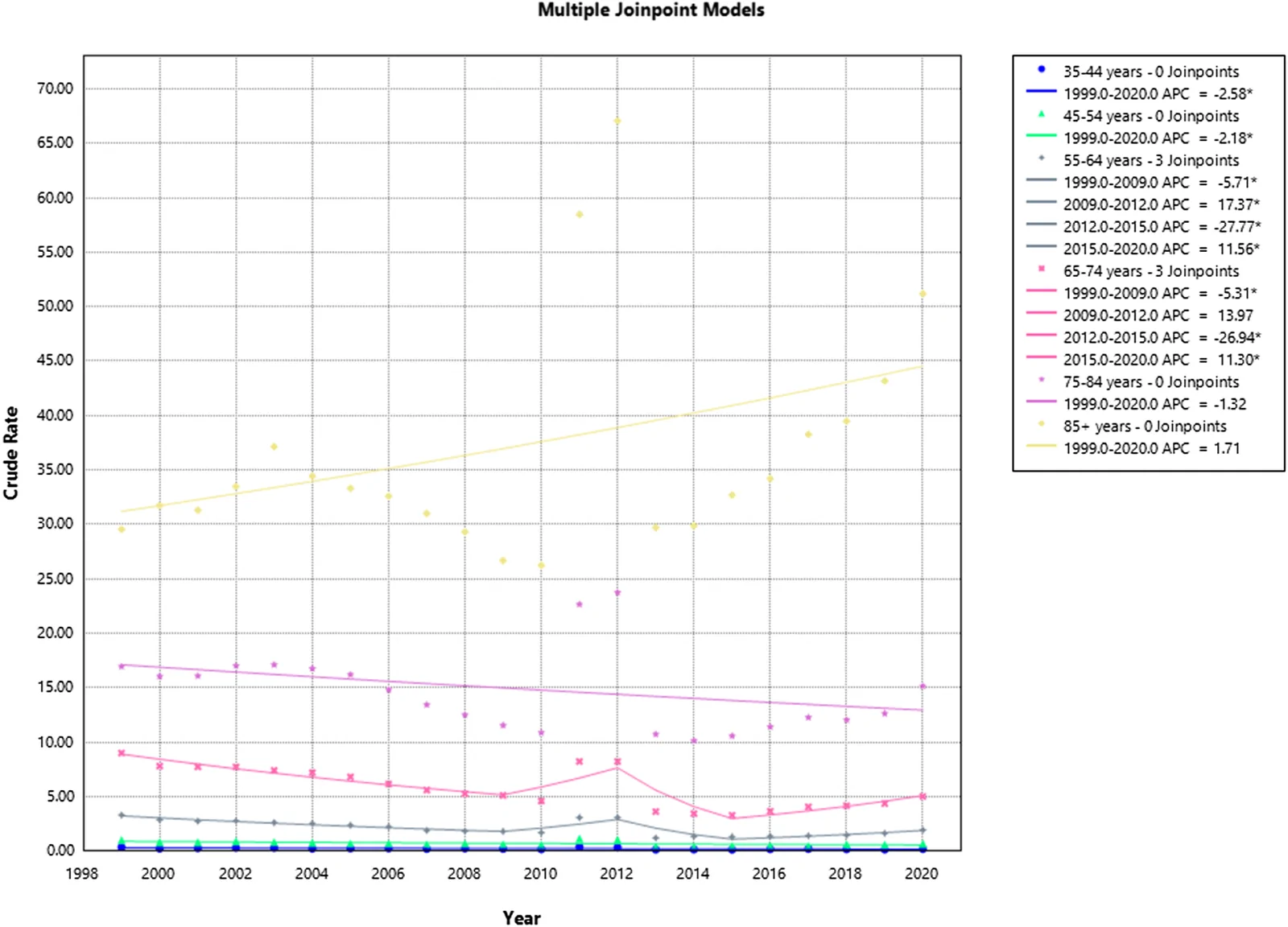
Age-stratified trends in age-adjusted mortality rates (AAMRs) per 100 000 individuals for stroke and chronic kidney disease in the United States from 1999 to 2020.

### Stroke and CKD-related AAMR stratified by geographic region

Large central metropolitan, medium metropolitan, small metropolitan, and micropolitan areas have similar AAMR values from 1999 to 2020 (70.05, 69.89, 70.59, and 70.22, respectively). Large fringe and noncore areas have AAMRs of 57.52 and 67.81, respectively (Supplemental Digital Content Table S6, available at: https://links.lww.com/MS9/B380).

Large central metropolitan areas showed an initial decrease from 1999 to 2009 (APC: −3.58; 95% CI: −5.16 to −1.96), followed by a sharp increase until 2012 (APC: 24.89; 95% CI: −0.62 to 56.96), then a steep decline until 2015 (APC: −29.59; 95% CI: −49.01 to −2.77), and finally a more moderate increase until 2020 (APC: 11.01; 95% CI: 4.90–17.47) (Fig. 6). Large fringe metropolitan areas showed an initial decrease from 1999 to 2009 (APC: −3.82; 95% CI: −4.88 to −2.73), followed by a sharp increase until 2012 (APC: 21.31; 95% CI: 2.75–43.22), then a steep decline until 2015 (APC: −24.91; 95% CI: −39.20 to −7.25), and finally a more moderate increase until 2020 (APC: 11.47; 95% CI: 7.52–15.56) (Fig. 6). Medium metropolitan areas showed an initial decrease from 1999 to 2009 (APC: −3.07; 95% CI: −4.53 to −1.57), followed by an increase until 2012 (APC: 22.40; 95% CI: −0.58 to 50.69), then a decline until 2015 (APC: −25.20; 95% CI: −43.25 to −1.41), and finally a moderate increase until 2020 (APC: 11.46; 95% CI: 6.52–16.62) (Fig. 6). Small metropolitan areas showed a nonsignificant average annual decrease across the entire period from 1999 to 2020 (APC: −1.00; 95% CI: −2.25 to 0.27). Micropolitan (nonmetro) areas showed a nonsignificant average annual decrease across the entire period from 1999 to 2020 (APC: −0.98; 95% CI: −2.13 to 0.18). Noncore (nonmetro) areas showed a nonsignificant average annual decrease across the entire period from 1999 to 2020 (APC: −0.92; 95% CI: −2.07 to 0.25) (Fig. 6). The most urban areas (large and medium metro) showed a highly volatile trend with a peak around 2012 and a steep decline by 2015. In contrast, the less urban and rural areas (small metro and nonmetro) maintained a stable, nonsignificant decrease over the entire period (Fig. 6).

**Figure 6.**
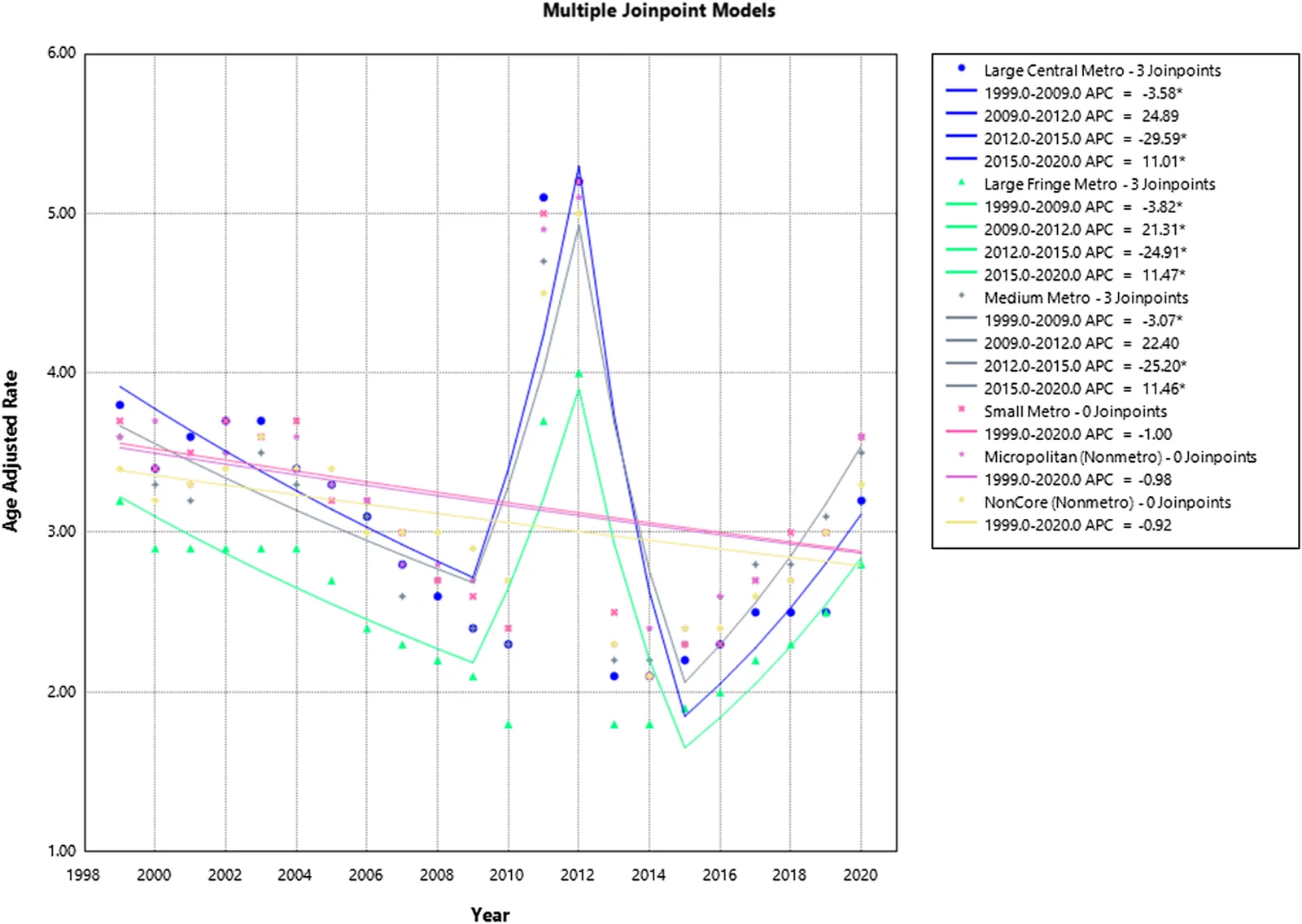
Urbanization-stratified trends in age-adjusted mortality rates (AAMRs) per 100 000 individuals for stroke and chronic kidney disease in the United States from 1999 to 2020.

Notable differences were observed in AAMR across the United States. The AAMR ranged from 4.28 in South Carolina to 1.45 in Arizona. States in the 90th percentile included South Carolina, West Virginia, North Carolina, North Dakota, the District of Columbia, and Maryland, with approximately twice the AAMR of states in the bottom 10th percentile, i.e., Montana, Utah, New York, Wyoming, Nevada, and Arizona (Fig. 7; Supplemental Digital Content Table S7, available at: https://links.lww.com/MS9/B380).

**Figure 7.**
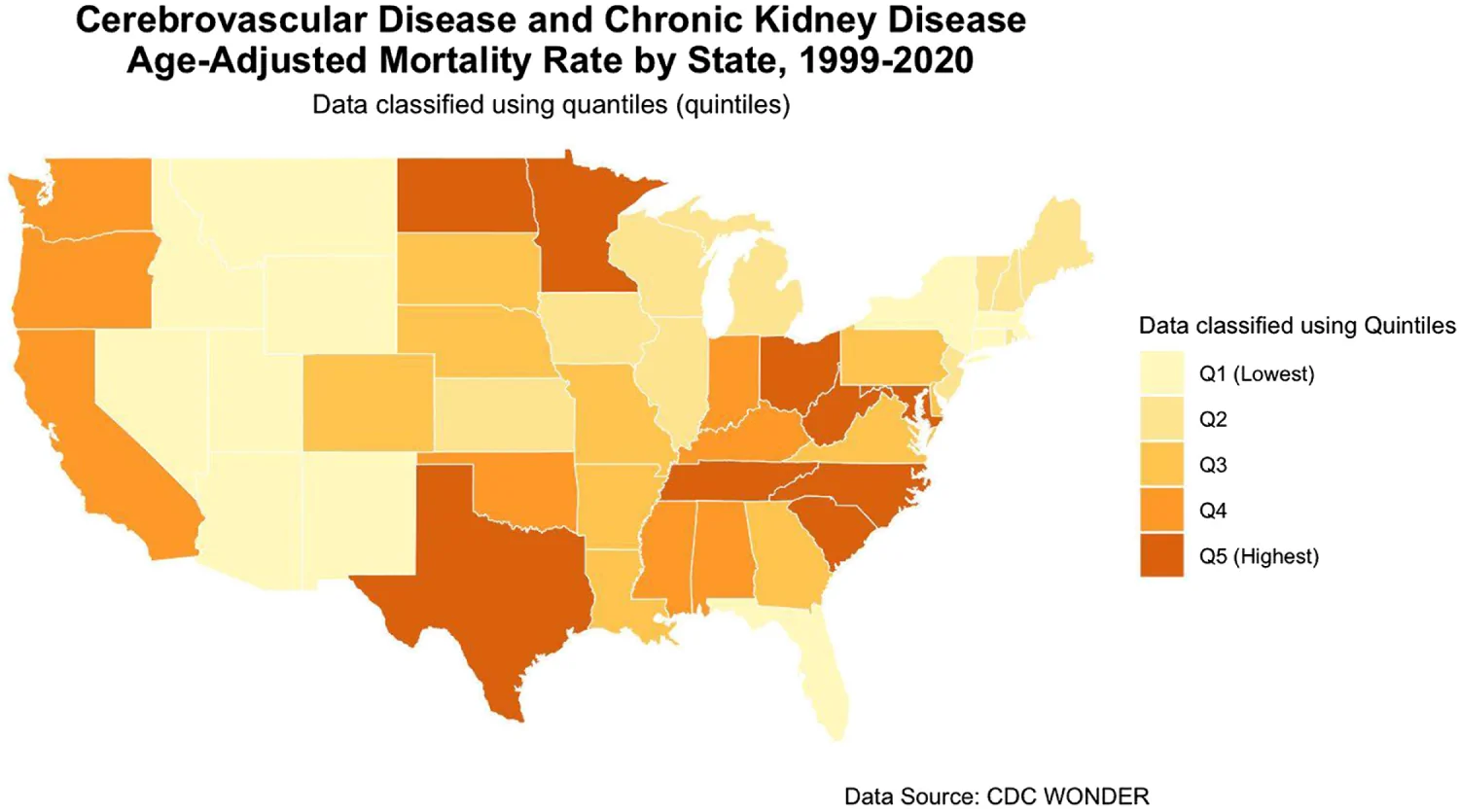
Age-adjusted mortality rates for cerebrovascular disease and chronic kidney disease by state, 1999–2020.

Looking at the census regions, the age-adjusted rate for the Northeast was 2.92 in 1999 and 2.38 in 2020 (Supplemental Digital Content Table S8, available at: https://links.lww.com/MS9/B380). It initially showed a significant decline for about a decade, from 1999 to 2009, with an APC of −4.74 (95% CI: −6.18 to −3.28). This was followed by a sharp surge from 2009 to 2012 (APC: 23.59; 95% CI: −0.03 to 52.80). The rate then experienced an equally swift and significant collapse from 2012 to 2015 (APC: −25.78; 95% CI: −44.41 to −0.91), before concluding with a moderate yet substantial upward trend from 2015 to 2020, with an APC of 11.45 (95% CI: 6.07–17.09) (Fig. 8).

**Figure 8.**
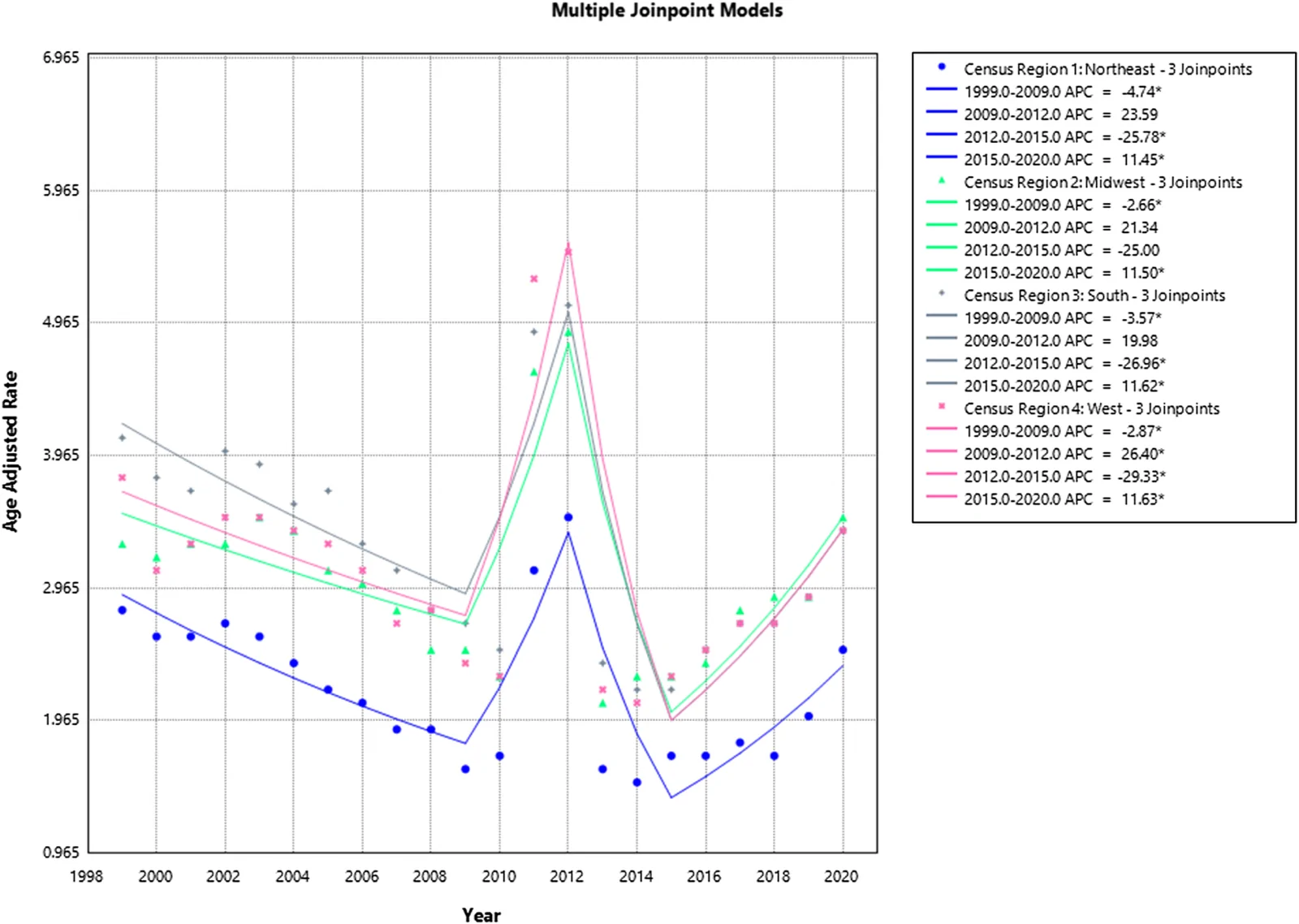
Census region-stratified trends in age-adjusted mortality rates (AAMRs) per 100 000 individuals for stroke and chronic kidney disease in the United States from 1999 to 2020.

The AAMR for the Midwest initially showed a significant decline from 1999 to 2009, with an APC of −2.66 (95% CI: −4.30 to −1.00). This was followed by a sharp, nonsignificant surge from 2009 to 2012 (APC: 21.34; 95% CI: −2.90 to 51.63), which was immediately succeeded by an equally swift, nonsignificant decline from 2012 to 2015 (APC: −24.99; 95% CI: −44.19 to 0.79). The period concluded with a powerful and statistically significant upward trend from 2015 to 2020, registering an APC of 11.50 (95% CI: 6.06–17.22) (Fig. 8). AAMR was 3.53 in 1999 and 3.50 in 2020 (Supplemental Digital Content Table S8, available at: https://links.lww.com/MS9/B380).

The South exhibited a statistically significant decrease across the first decade, from 1999 to 2009, with an APC of −3.57 (95% CI: −4.93 to −2.19). This initial trend was abruptly interrupted by a sharp, though nonsignificant, increase from 2009 to 2012 (APC: 19.98; 95% CI: −1.76 to 46.53). Subsequently, the rate experienced a decline between 2012 and 2015, dropping by an APC of −26.96 (95% CI: −44.49 to −3.88). The trend concluded with a highly significant reversal and sustained ascent from 2015 to 2020, with the rate climbing at an APC of 11.62 (95% CI: 6.31–17.19) (Fig. 8). The AAMR was 4.21 in 1999 and 3.41 in 2020 (Supplemental Digital Content Table S8, available at: https://links.lww.com/MS9/B380) The data for the West reveal a significant decline from 1999 to 2009, where the rate fell by an APC of −2.87 (95% CI: −4.44 to −1.27). This was immediately followed by a statistically significant increase between 2009 and 2012, with an APC of 26.40 (95% CI: 1.21–57.86). The rate then experienced an equally sharp and significant plunge from 2012 to 2015, with an APC of −29.33 (95% CI: −48.10 to −3.78). The period concluded with a highly significant rebound and sustained increase from 2015 to 2020, with an APC of 11.63 (95% CI: 6.04–17.50) (Fig. 8). The AAMR was 3.69 in 1999 and 3.41 in 2020 (Supplemental Digital Content Table S8, available at: https://links.lww.com/MS9/B380).

## Discussion

To the best of our knowledge, this study is the first to offer a comprehensive analysis of stroke- and CKD-related mortality stratified by demographic factors and regions across the United States from 1999 to 2020. Over this period, a total of 140 501 stroke- and CKD-related deaths were reported. There was an overall decrease in mortality rates from 1999 to 2020. This might be due to several factors, such as medical advancements, technological innovations, and national programs aimed at increasing awareness of cardiovascular risk factors. This decrease may also be due to the effective control of hypertension, which has lowered both stroke incidence and case-fatality rates. There have also been improvements in diabetes, lipid, and smoking-cessation interventions^[^16^]^.

The majority of deaths occurred among females, yet they had a lower AAMR than males. This contrasts with a retrospective study conducted in the United States, where males had a higher number of deaths than females and also a higher AAMR^[^17^]^. The higher number of deaths in females might be because they make up a larger share of the elderly population, and mortality from CKD and stroke is higher in elderly women. A review stated that women exhibit a higher or comparable stroke incidence compared with men in younger age groups^[^18^]^. During middle age, stroke incidence becomes higher among men^[^18^]^. However, as women reach the postmenopausal period, this sex difference narrows, and by their 80s, the incidence in women approaches or even exceeds that of men^[^19^]^. The same review also stated that a lower age-adjusted incidence of stroke in women than in men has been consistently reported^[^18^]^. Nonetheless, there was an overall decrease in AAMRs in both males and females from 1999 to 2020.

Individuals with CKD face a significantly higher risk of both ischemic and hemorrhagic strokes. CKD increases stroke risk through multiple mechanisms, including vascular calcification, arterial stiffness, chronic inflammation, and impaired regulation of cerebral blood flow. In patients with advanced CKD or those on dialysis, these factors, along with uremia and anticoagulation-related bleeding risk, further elevate the likelihood of both ischemic and hemorrhagic strokes^[^20^]^. Effective control of blood pressure is crucial in reducing stroke risk among CKD patients^[^21,22^]^.

Black or African American individuals had the highest AAMR but also showed the most significant decline, whereas White individuals had the lowest AAMR with minimal change. Structural racism, socioeconomic inequalities, and limited access to care and neighborhood resources contribute to higher burdens of social determinants of health in Black populations, which help explain their elevated mortality (AAMR) compared with White individuals ^[^23^]^. The higher AAMR in Black individuals is likely due to the greater prevalence and impact of hypertension and diabetes, while their significant decline reflects improvements in the management of these risk factors^[^24^]^. The second-highest AAMR was in the Hispanic or Latino population. This poor health outcome may be due to limited access to health insurance and preventive care, employment in high-risk occupations, cultural barriers, racism, and communication barriers^[^23,25^]^.

All four US census regions initially saw a decline in age-adjusted mortality, followed by a brief surge, then a subsequent decline, with recent years showing a sustained upward trend. While the timing was similar, the magnitude of changes varied slightly between regions. The Northeast has a lower AAMR than the other regions. This is consistent with a similar study^[^9^]^. States in the 90th percentile had approximately double the AAMR compared with those in the 10th percentile. The same study also reported similar results^[^9^]^. The Stroke Belt phenomenon seems to apply here too, as the states in those regions are in the top 90th percentile. The findings from Douthit *et al* (2015) highlight the importance of addressing healthcare access disparities to improve health outcomes^[^26^]^. The observed urban–rural differences may reflect variations in healthcare access, socioeconomic conditions, and population risk profiles. Urban areas likely experienced greater fluctuations due to rapid demographic and lifestyle changes, while rural regions showed steadier trends, possibly indicating slower improvements in prevention and healthcare delivery. The differences in AAMRs across race, ethnicity, and geography in our study show disparities among these groups. Another study also highlighted emerging disparities by race, ethnicity, and geography^[^27^]^.

Mortality was lowest in younger adults and highest among those aged 85 years and above. The CMR was high in older adults, especially those aged 65 years and above. This suggests that older adults with CKD remain at a high and persistent risk of stroke mortality. The risk of stroke occurrence is higher in older adults with CKD, and this elevated susceptibility likely contributes to the higher stroke-related mortality observed in this population^[^2^]^. Age-related vascular changes and accumulated comorbidities further exacerbate both the incidence and severity of strokes in these patients^[^28^]^.

## Limitations

The analysis is predicated on death certificate data from the CDC WONDER database, which may be incomplete or misclassified in terms of cause of death. Accurate identification of stroke and CKD depends on certifying physicians correctly assigning ICD-10 codes (I60–I69 for stroke and N18 for CKD). This coding could change over time and between states. The patient’s socioeconomic status, healthcare access, treatment type, and method of diagnosis are not disclosed by CDC WONDER. This study, being retrospective and based on aggregate population-level data, cannot establish causal relationships or control for potential confounding factors. There is no information on patients’ comorbidities, risk factors, or laboratory results. Among 140 501 deaths, data on place of death were accessible for 140 144 deaths. The remaining data were suppressed, most likely for confidentiality reasons. This suppression limited our ability to evaluate differences by place of death. CDC WONDER reports only CMRs when data are stratified by age groups or place of death, and it does not provide AAMRs. Also, urban–rural and regional classifications were based on the 2013 NCHS and US Census definitions, which may not perfectly reflect population changes or urbanization patterns during the entire 1999–2020 period.

## Conclusion

Stroke- and CKD-related mortality in the United States declined overall between 1999 and 2020. Despite this progress, disparities remain across sex, race, age, and geography. Targeted interventions are needed to reduce these persistent demographic and regional disparities.

## Data Availability

The datasets used during this study will be available from thecorresponding author upon reasonable request.
